# Ultrasmall Au and Ag Nanoclusters for Biomedical Applications: A Review

**DOI:** 10.3389/fbioe.2020.01019

**Published:** 2020-10-08

**Authors:** Jia Tang, Haihong Shi, Guanyu Ma, Liangping Luo, Zhenghua Tang

**Affiliations:** ^1^Medical Genetics Center, Jiangmen Maternity and Child Health Care Hospital, Jiangmen, China; ^2^Guangzhou Key Laboratory for Surface Chemistry of Energy Materials, Guangzhou Higher Education Mega Centre, School of Environment and Energy, New Energy Research Institute, South China University of Technology, Guangzhou, China; ^3^Department of Medical Imaging Center, The First Affiliated Hospital of Jinan University, Jinan University, Guangzhou, China; ^4^Guangdong Engineering and Technology Research Center for Surface Chemistry of Energy Materials, Guangzhou Higher Education Mega Centre, School of Environment and Energy, South China University of Technology, Guangzhou, China

**Keywords:** ultrasmall Au and Ag nanoclusters, biomedical applications, bioimaging and biosensing, anti-microbial applications, tumor targeting and cancer treatment

## Abstract

Noble metal (e.g., Au, Ag, Pt, Pd, and their alloys) nanoclusters (NCs) have emerged as a new type of functional nanomaterial in nanoscience and nanotechnology. Owing to their unique properties, such as their ultrasmall dimension, enhanced photoluminescence, low toxicity, and excellent biocompatibility, noble metal NCs—especially Au and Ag NCs—have found various applications in biomedical regimes. This review summarizes the recent advances made in employing ultrasmall Au and Ag NCs for biomedical applications, with particular emphasis on bioimaging and biosensing, anti-microbial applications, and tumor targeting and cancer treatment. Challenges, including the shared and specific challenges for Au and Ag NC toward biomedical applications, and future directions are briefly discussed at the end.

## Introduction

The last decade has witnessed the great achievement of employing noble metal nanoparticles, with sizes typically between 2 and 100 nm, in biomedical applications (Li et al., 2017; Mohanta et al., 2020; Yang et al., 2020). Such applications are largely relied on for their optical properties, such as absorption, luminescence, surface scattering, and surface-enhancing capabilities. For instance, spherical Au nanoparticles (NPs) of ∼5–100 nm often display a strong absorption peak at ∼520 nm, otherwise called the surface plasmon resonance (SPR) peak, which can be fine-tuned by manipulating the size and morphology of the Au NPs. The SPR peak originates from the collective excitation of free electrons upon excitation by light (Murray, 2008; Chakraborty and Pradeep, 2017).

However, when the size of metal NPs shrink below 2 nm, the SPR peak disappears and discrete step-like absorption bands can be observed in the optical spectrum. Such ultrasmall NPs are called nanoclusters (NCs), and NCs exhibit significantly different physiochemical properties from relatively larger counterparts, such as having larger Stokes shift, enhanced fluorescence, excellent photostability, and so on (Tan and Jin, 2013; Chakraborty and Pradeep, 2017). In addition, by controlling the reaction kinetics, it is possible to prepare ultrasmall NCs with atomic precision, where the definitive composition and molecule-like properties can provide an ideal bridge to establish the structure/function relationship for nanoscience and nanotechnology research, which are not achievable for NPs because of their polydisperse nature in terms of size, morphology, and structure (Tang et al., 2018; Du et al., 2020).

For biomedical applications, metal NCs have demonstrated some superior performances over their NP counterparts. For instance, when utilized in an *in vivo* setup, metal NPs couldn’t escape from the kidney barrier, which might cause some severe side effects in the liver and spleen, but in stark contrast, the ultrafine size of metal NCs allow them to be efficiently cleared from the body without causing serious damage (Song et al., 2016). In biomedical research, it is highly desirable to have a reliable, sensitive, and biocompatible platform, and the materials employed are expected to be stable and to maintain their intrinsic properties. The target matrix, *in vivo* environment are also complex, therefore the capability to fine-tune the physiochemical properties of the materials without sacrificing the integrity of nanomaterials is critical, where the surface chemistry of NCs are beneficial to achieve this goal (Song et al., 2016).

The controllable synthesis, catalytic applications, and surface chemistry engineering of noble metal clusters, especially Au and Ag NCs, have been discussed in some recent reviews (Li and Jin, 2013; Chakraborty and Pradeep, 2017; Higaki et al., 2018; Tang et al., 2018; Yan et al., 2018; Kang and Zhu, 2019; Du et al., 2020). However, the recent advances of employing ultrasmall Au and Ag NCs for biomedical applications have not before been summarized. This review summarizes the recent progresses made in engineering Au and Ag NCs for biomedical applications, with particular emphasis placed on bioimaging and biosensing, anti-microbial applications, and tumor targeting and cancer treatment. In the conclusion, the challenges, including the shared and specific challenges for Au and Ag NC toward biomedical applications, and future research directions in this emerging and promising field are discussed.

## Ultrasmall Au and Ag NCs for Biomedical Applications

### For Bioimaging and Biosensing

NCs are ultrasmall NPs; as the size approaches the Fermi-wavelength of the electrons, metal NCs exhibit molecule-like characteristics (Murray, 2008). For example, metal NCs always have discrete absorption energy states, excellent photostability, and strong luminescence, which is an intriguing property which makes them immediately suitable for biomedical research (Zheng et al., 2017b). Such luminescence produces a large Stokes shift and a long lifetime, and the photoluminescence wavelengths of NCs can be tuned from near infrared to ultraviolet by manipulating their size and composition. Three important features of NCs mainly contribute to the photoluminescence, namely the quantum size effects, the nature of the ligand, and the aggregation of the gold species and the aurophilic interactions (Li et al., 2020).

Early work that utilized the photoluminescence of Au NCs in the biomedical field mainly focused on bio-imaging. For instance, Conroy discovered that, through thermal treatment, the near infrared luminescence of mercaptosuccinic acid and tiopronin-protected Au NCs were drastically enhanced, probably due to the unstable non-luminescent molecules being transformed into stable luminescent clusters with narrow size distributions (Conroy et al., 2014). Meanwhile, PEGylation of the Au NCs was able to improve the permeation into the cytoplasm, and these PEGylated Au NCs can penetrate inside the cell nucleus in single cell imaging (Zhang et al., 2012; Conroy et al., 2014). Wang and Shao’s groups fabricated l-carnosine protected fluorescent Au NCs, which exhibited bright blue photoluminescence and low toxicity for HeLa cell imaging (Li et al., 2016). In a recent report, Liu et al. prepared an atomic-precision Au cluster with 25 gold atoms and 18 peptide ligands, and such a cluster could serve as an NIR-II fluorophore with emission at 1,100–1,350 nm (Liu et al., 2019). The Au NCs based NIR-II imaging was able to monitor many small vessels thanks to the enhanced permeability of brain vessels after a stroke. It is conceivable that Au NCs can serve as NIR-II dye for biological imaging, especially brain imaging.

**FIGURE 1:**
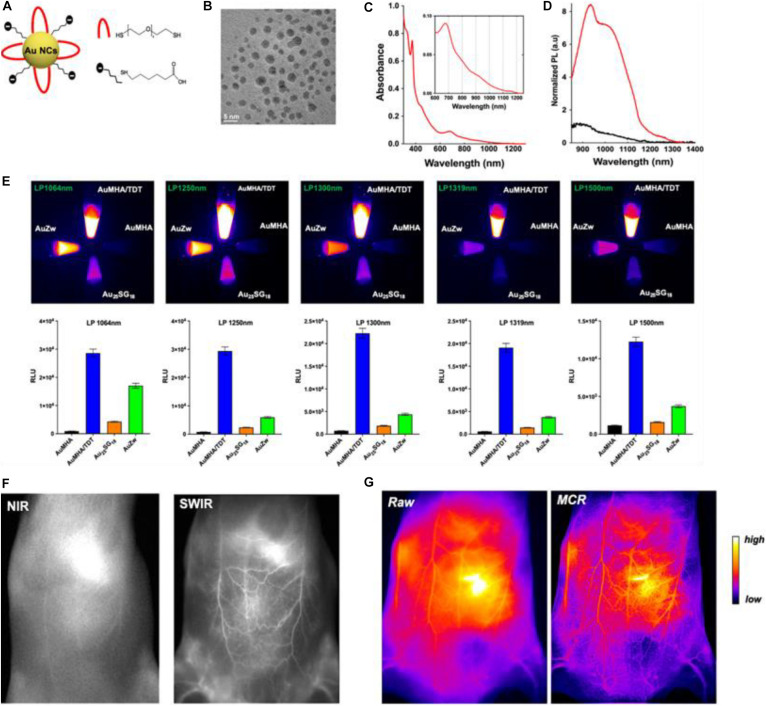
**(A)** Scheme of the Au NCs AuMHA/TDT (MHA: black; TDT: red). **(B)** HR-TEM image of AuMHA/TDT. **(C)** Absorbance spectrum of AuMHA/TDT. **(D)** PL spectra of AuMHA (black line) and AuMHA/TDT (red line). **(E)** SWIR PL of AuMHA, AuMHA/TDT, Au_2__5_SG_18_, and AuZW under NIR excitation. **(F)** NIR I and SWIR imaging of mice 15 min after injection of AuMHA/TDT. **(G)** Non-invasive imaging of the mouse ventral area before (raw) and after MCR processing. Reproduced with permission. Copyright 2020, American Chemical Society.

Recently, Yu et al. (2020) reported on the fabrication of water-soluble Au NCs which are protected by co-ligands, including a short dithiol pegylated chain (**AuMHA/TDT**). Intriguingly, the AuMHA/TDT sample exhibited a high brightness in the shortwave infrared (SWIR) spectrum with a detection above 1,250 nm. Figure 1A shows that the Au NCs were synthesized by using mercaptohexanoic acid (MHA) and tetra(ethylene glycol) dithiol (TDT) as co-ligands, while Figure 1B illustrates that they are monodispersed with an average diameter of 2.1 ± 0.6 nm. The dithiol ligand of TDT led to the presence of NIR absorbance features at 800, 910, and 1,140 nm (Figure 1C), while the anisotropic surface of Au NCs produced a 9-fold increase of photoluminescence in a longer wavelength range (Figure 1D). When using long-pass filters above 1,250 nm, AuMHA/TDT exhibited a 12-fold more intense signal, as manifested by the images in Figure 1E. Remarkably, the AuMHA/TDT had a quantum yield of ∼6%, higher than the other reported molecular Au NCs such as **Au25(SG)18** (Liu et al., 2019). It can be noted that there is a dramatic improvement of the spatial resolution in the SWIR, as compared to the NIR I (Figure 1F). The authors also conducted the Monte Carlo constrained restoration (MCR) method to further improve the spatial resolution and overcome the scattering from the skin and the tissues. Such imaging processing greatly enhanced the spatial resolution, as observed in Figure 1G. This study highlights the Au-NCs-based SWIR-emitting contrast agent for bio-imaging and other advanced bio-medical applications (Yu et al., 2020). However, such SWIR properties can only be achieved through rational ligand design, and the quantum yields still need to be improved.

Besides bio-imaging, Au NCs can also be utilized for biosensing. For example, Biswas et al. (2017) developed a new type of bovine serum albumin-capped Au nanoclusters (BSA-AuNCs) containing polymeric microcapsules as a H_2_O_2_ sensor, where the change in H_2_O_2_ within living cells can be visualized and intracellular H_2_O_2_ fluctuations in response to external stimuli were monitored. Such BSA-AuNCs possessed near infrared emitting behaviors, and when conjugated with chemically modified cellulose strips, they showed a nanomolar detection limit of mercuric ions (Bothra et al., 2017). Other biomolecules, such as peptide and protein, can also be used as capping agents to prepare fluorescent Au NCs toward sensing applications. Hossein-Nejad-Ariani et al. (2018) developed a peptide-capped Au-NCs-based biosensor for the detection of Listeria monocytogenes, which is portable, simple, fast, and can be operated by non-experts. Akyüz et al. (2019) fabricated chicken egg white protein capped Au NCs as a biosensor, which was able to determine the pro-oxidant activity of natural antioxidant compounds. Electrochemiluminescence (ECL) is another type of chemiluminescence, and the Au NCs hold the ECL properties that are not available from **Au NPs (**Wang et al., 2016; Chen et al., 2019). The ECL behaviors can also be designed for biosensing; for example, Zhang et al. reported that the ultrasensitive signal for detecting acetylthiocholine can be recorded on an Au-NCs-based ECL biosensors (Zhang et al., 2019).

Despite the relatively lower photostability toward oxidation of Ag, previous reports have shown that Ag NCs with the same ligand could have very intense fluorescence signals as well (Tao et al., 2015; Song et al., 2016). In an early study done by Le Guével et al. (2012), glutathione (GSH)-protected Ag NCs possessed sizes smaller than 2 nm and the yellow-emitting Ag NCs had a quantum yield over 65%, and these Ag NCs were employed as fluorescent labels to visualize A549 cells. Interestingly, human hemoglobin (Hb) can be a stabilizer, reducer, and linker to synthesize Hb-AgNCs with single excitation and dual maximum emissions by a facile one-pot green approach without using toxic reductants such as **NaBH4 (**Shamsipur et al., 2018). The aggregation of oligomeric Ag(I)-Hb intermediates caused aggregation-induced emission (AIE), and such emissions were used as selective probes for HeLa cell imaging with high bio-compatibility and specificity (Shamsipur et al., 2018).

Fluorescent Ag NCs have also demonstrated great promise as substitutes for conventional probes and sensors. Yeh group reported the activatable and color-switchable properties of DNA-templated Ag NCs for DNA sensing (Obliosca et al., 2013). Yuan et al. developed a simple approach to detect cysteine with high sensitivity and selectivity by using highly luminescent Ag NCs, which showed superior selectivity for cysteine over the other 19 natural non-thiol-containing amino acids (Yuan et al., 2013b). Pan et al. (2018) fabricated a new paradigm for label-free amplified nucleic acid detection by combining the highly efficient signal-amplification capability of catalytic hairpin assembly (CHA) reaction with the spatially sensitive fluorescent Ag NCs, which can be integrated as an intact and smart apparatus.

**FIGURE 2:**
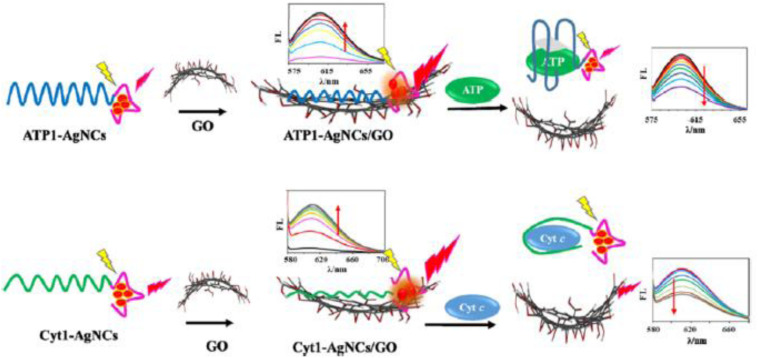
Working principle of the label-free ATP and Cyt c sensing based on DNA-stabilized AgNCs/GO nanohybrids. Reproduced with permission, Copyright 2019, American Chemical Society.

Recently, Shamsipur et al. (2019) reported a new AIE-active luminescent sensor based on DNA-AgNCs/GO (graphene oxide) nanohybrids for the detection of triphosphate (ATP) and cytochrome (Cyt) c in cell lysates. The working principle is shown in Figure 2. In the absence of a target, the DNA-AgNCs are adsorbed onto the GO surface, while the fluorescence is enhanced because of the AIE enhancement characteristic of DNA-AgNCs when they are adsorbed onto the GO surface. Upon target binding, the conformation of specific aptamers is significantly altered and AgNCs are desorbed on the GO surface, leading to the restoration of the fluorescence signal (Shamsipur et al., 2019). This study opens a new avenue for designing noble metal NCs-based AIE sensors, where the performance stability of such Ag NCs-based AIE sensors must be further examined in future studies.

### Anti-microbial Applications

Metallic Ag has been used to combat infection since ancient times (Rai et al., 2009). With the great advancements made in nanomaterial and nanotechnology, Ag’s efficacy to fight wide-spectrum infections has been significantly improved in the form of Ag NPs (Wijnhoven et al., 2009; Marambio-Jones and Hoek, 2010). Thanks to the unique chemistry of Ag interacting with microorganisms, Ag can exert a broad-spectrum of antimicrobial properties through several killing mechanisms, including membrane damage, DNA destruction, and, particularly, generating reaction oxygen species (ROS) to perturb cell metabolism (Xiu et al., 2012; Chernousova and Epple, 2013; Rizzello and Pompa, 2014).

It is conceivable that when Ag NPs downsize the diameter to a sub-nanometer regime, the antibacterial efficacies of Ag can be significantly increased due to the much higher surface-to-volume ratio. Additionally, these Ag NCs with well-defined structures can produce other advantages, such as facile post-functionalization, good stability, and excellent and tunable photoluminescence, not to mention that the definitive composition and structure can provide an ideal platform to establish the correlation between the anti-microbial activities of the Ag NCs and their structures. Yuan et al. (2013a) synthesized a series of water-soluble thiolate Ag NCs with strong luminescence and tunable emissions, and these NCs possessed superior antimicrobial properties against the multidrug-resistant bacteria Pseudomonas aeruginosa by generating a high concentration of intracellular ROS. In another study, they discovered that different charge states can significantly affect the anti-microbial properties. The antimicrobial tests showed that Ag^+^-enriched NCs had much higher antimicrobial activity than metallic Ag NCs for both gram negative (i.e., *P. aeruginosa* and *E. coli*) and gram positive (i.e., *B. subtilis* and *S. aureus*) bacteria (Yuan et al., 2014). Wang et al. (2020) showcased that the ultrasmall Ag NCs can be embedded in Luria-Bertani extract via light irradiation, and enhanced antibacterial activity with good bio-compatibility were achieved. Recently, Liu et al. (2020) reported on an Ag-NC-based hydrogel that exhibited a superior, broad spectrum antimicrobial performance against both gram-negative and gram-positive bacteria. It also demonstrated long-acting bactericidal efficacy compared with pristine Ag NCs, mainly thanks to its controllable release capability for Ag species.

**FIGURE 3:**
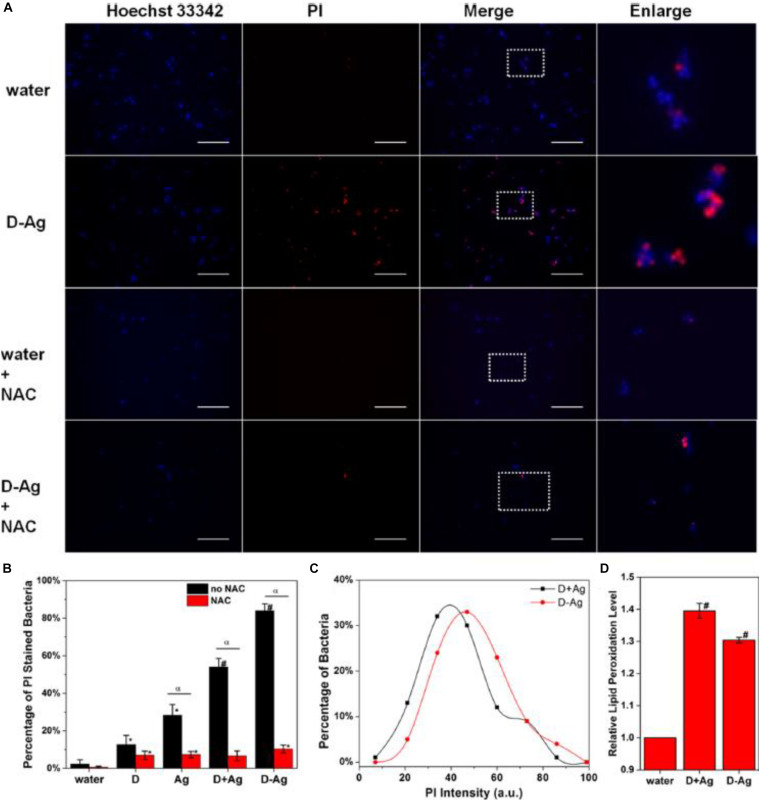
**(A)** Representative fluorescence images of the bacterial cells after 2 h treatment. **(B)** Percentage of PI-stained bacteria to show damaged bacterial membrane. * is significant against the water treated group, *p* < 0.05; # is significant against the water-treated group, *p* < 0.001. α is significant difference against non-NAC-treated group, *p* < 0.05. **(C)** Histogram of the PI intensity in each individual bacterial cell treated with D + AgNCs (black line) and D-AgNCs (red line). **(D)** Relative lipid peroxidation level after incubation for 2 h, where the lipid peroxidation of the water-treated group was set as 1. # is significant against the water treated group, *p* < 0.001. Reproduced with permission, copyright 2016, American Chemical Society.

More intriguingly, Ag NCs can be integrated with other bactericide to form an entity, which can act as a promising platform to improve the efficiency of antimicrobial agents. For instance, Zheng et al. (2016) designed an antimicrobial hybrid by conjugating molecular Ag_1__6_(SG)_9_ clusters with daptomycin (D-AgNCs), and such a hybrid showed improved bacterial killing efficiency over the physically mixed Ag NCs and daptomycin (D + AgNCs), mainly because the D-Ag NCs were able to effectively damage the bacterial membrane. The ROS was involved in destroying the bacteria wall, while N-acetyl-Lcysteine (NAC) could decrease the efficiency of Ag NCs by mitigating the ROS generated by Ag NCs. As illustrated in Figure 3A, NAC significantly inhibited the membrane damage. Figure 3B further provides supportive evidence that ROS was involved in the membrane damage induced by D-AgNCs. Figure 3C shows that the bacterial cells treated by D-AgNCs exhibited a right shift of propidium iodide intensity compared with D + AgNCs, indicating more PI were present inside the bacteria treated by D-AgNCs. Finally, Figure 3D demonstrates that D-AgNCs had a higher degree of lipidc peroxidation than D + AgNCs, implying that more ROS was generated by D-AgNCs (Zheng et al., 2016). Such an integrating strategy with other bactericide might be applicable for other antimicrobial applications, but the exact working mechanism of such an entity still needs to be further elucidated.

Bulk gold is known to be chemically inactive, however, when the size of Au decreases to nanoscale dimensions or even close to 1 nm or the sub-nanometer regime to form Au NPs and NCs, interesting physiochemical properties appear in Au NPs, and especially in Au NCs. These intriguing properties could spawn a wide spectrum of applications in various fields, including in biomedical research. For instance, by grafting known antimicrobial compounds, such as ampicillin, antimicrobial peptides, and cationic or zwitterionic ligands, on the surface of Au NPs, Au NPs can act as passive drug carriers and exhibit some outstanding antimicrobial behaviors (Li et al., 2014; Huo et al., 2016; Kuo et al., 2016; Rai et al., 2016). Since enhanced anti-microbial activities can be obtained when the size of Ag NPs was reduced to the NC range, it is conceivable that the Au NCs also exhibit some bacteria killing properties. Indeed, Zheng et al. (2017a) found that ultrasmall Au NCs showed high broad-spectrum antimicrobial activities, which is actually absent in the Au NPs counterparts protected by the same ligand. Interestingly, Yang’s group discovered that the translocation of Au NCs into non-photosynthetic bacteria could enable the photosynthesis of acetic acid from CO_2_, and such photogenerated CO_2_ fixation was able to operate continuously for several days (Zhang et al., 2018).

**FIGURE 4:**
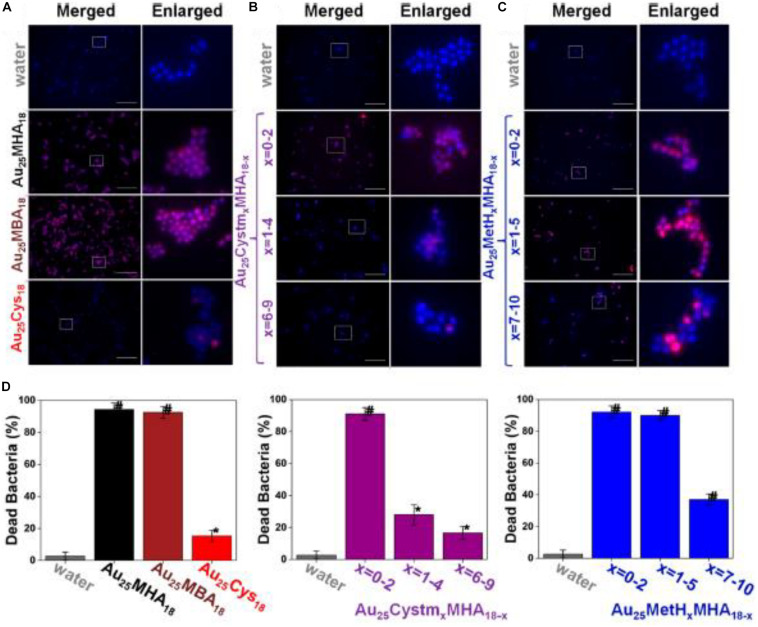
Au_25_ NCs protected by thiolate ligands bearing a higher ratio of –CO_2_H groups showed better bacterial killing efficiency. **(A–C)** Merged and enlarged representative fluorescence images of the *S. aureus* after 2 h treatment of Au_25_ NCs. **(D)** Percentage of the dead *S. aureus* after being treated with different ligand-protected Au_25_ NCs for 2 h. *Significant against the water-treated group, *p* < 0.05; ^#^Significant against the water-treated group, *p* < 0.001. Reproduced with permission, copyright 2018, American Chemical Society.

Another intriguing advantage for employing ultrasmall Au NCs for anti-microbial applications is that the surface of the Au NCs can be manipulated, which provides a paradigm to modulate the efficacy of Au NCs. By leveraging the molecular properties of ultrasmall Au NCs, the surface properties of the thiolate Au NCs were able to be precisely controlled at an atomic level. Zheng et al. (2018) fabricated a family of Au NCs with identical Au atoms in the core but different properties. Five different Au_25_ NCs with different ligands were synthesized, and the bacteria killing data are presented in Figure 4 (Zheng et al., 2018). As illustrated in Figure 4A and left columns in Figure 4D, the Au_2__5_(MHA)_18_ NCs (MHA = 6-mercaptohexanoic acid) effectively killed ∼95% of *S. aureus*, and Au_2__5_(MBA)_18_ (MBA = 3-mercaptobenzoic acid) which carries the similar monotypic functional group of –CO_2_H, exhibited a similar killing effect, where ∼93% of *S. aureus* population was effectively terminated. In stark contrast, Au_2__5_(Cys)_18_ (Cys = cysteamine hydrochloride) NCs drastically lost the effective antimicrobial properties, and only ∼15% of the *S. aureus* population was dead. An inverse relationship can be observed between the number of –NH_2_ group on the Au_25_ NCs surface and the anti-microbial efficacy. As shown in Figure 4B and middle columns of Figure 4D, the more Cystm (Cystm = cysteamine hydrochloride) being capped on the NC surface, the lower the antimicrobial ability the NCs had. It suggests that, for Au_2__5_(Cys)_18_, more –NH_2_ and less –CO_2_H could nullify the bacterial killing efficacy of the NCs. By incorporating MetH (MetH = 2-mercaptoethanol) on the NC surface, the resultant Au_2__5_(MetH)_*x*_(MHA)_18__–__*x*_ could carry both –CO_2_H and –OH groups on the NCs surface. Interestingly, the impact of the –OH group toward the killing efficiency of Au_25_ NCs was not as remarkably decreased as those with the –NH_2_ group, as illustrated in Figure 4C and the right columns of Figure 4D. Overall, more negatively charged Au NCs would generate more ROS, leading to a better bacterial killing efficiency (Zheng et al., 2018). This study showed the results are different from the common belief that positively charged Au NCs have better antimicrobial properties, but whether it is a special case still need to be explored. However, one can notice that the surface ligands are playing critically different roles in determining the antimicrobial behaviors, which opens an avenue for further development.

### Tumor Targeting and Cancer Treatment

Thanks to the easy surface functionalities and their excellent optical properties, especially their intriguing luminescent behaviors, ultrasmall Au and Ag NCs have shown great potential for therapeutic applications toward tumor cell targeting and cancer treatment (Song et al., 2016; Zhang et al., 2020). To target cancer cells, biocompatible ligands, such as GSH, are normally employed. In 2014, Zhang et al. (2014a) groups reported the construction of a new type of radiosensitizer by integrating Au_25_ NCs with GSH and bovine serum albumin (BSA). Thanks to the strong radiotherapy enhancement from the Au core, the ultrasmall Au_25_ NCs capped by both GSH and BSA demonstrated superior tumor accumulation via the improved enhanced permeability and retention (EPR) effect, resulting in a stronger enhancement for cancer therapy than the much larger Au NPs. Such an enhancement can be probably attributed to the photoelectric effect and Compton scattering of Au_25_ NCs, where GSH-capped Au_25_ NCs exhibited a remarkable decrease in tumor volume and weight as the radio sensitizer. Moreover, these clusters also exhibited very efficient renal clearance and had no obvious toxicity in the body, while in sharp contrast, BSA capped Au_25_ NCs were not able to be completely removed by the kidney and caused liver damage (Zhang et al., 2014a). Smaller Au NCs showed even better therapeutic effects. Zhang et al. (2014b) discovered that the ultrasmall sized clusters of Au_1__0–12_(SG)_1__0–1__2_ could increase the tumor uptake and the targeting specificity through the EPR effect, while the biocompatible GSH ligand with high exposure could further promote the tumor uptake by allowing the nano-molecules to escape the reticulo-endothelial system (RES) and activating the transporter on the cell surface.

The above strong size-dependent tumor targeting and renal clearance effects were further verified by Zheng’s group with a series of few-atom AuNCs (Du et al., 2017). In this study, a series of atomically precise glutathione-coated AuNCs were prepared (Du et al., 2017). At 40 min upon injection into mice, much more Au_1__0–1__1_ and Au_18_ NCs were retained in the kidneys than Au_25_ NCs. In addition, the ratios of bladder to kidney intensity derived from the X-ray imaging followed an order of Au_25_ NCs > Au_18_ NCs > Au_1__0–1__1_ NCs. As the Au NCs are highly physiologically stable and resistant to serum protein adsorption in both short and long periods, such differences in bladder/kidney ratios strongly suggest that the kidney filtration is highly sensitive to the cluster size. Au_25_ NCs had the best clearance efficiency, comparable to the ∼1.7 nm GSH-protected Au NPs (GS-AuNPs, average composition: ∼Au201) (Zhou et al., 2011), while the Au_1__0–1__1_, Au_15_, and Au_18_ NCs had efficiencies between the 2.5 nm GS-AuNPs (Average composition: ∼Au640) (Zhou et al., 2012) and 6 nm GS-AuNPs (Average composition: ∼Au_8856_), in which only about 4% could be cleared out of the body in 24 h (Zhou et al., 2011). In fact, it implies that the glomerulus, composed of glycocalyx, endothelial cells, glomerular basement membrane (GBM), and podocyte, is actually no longer a one-directional “size-cutoff” slit, but has become an atomically precise “bandpass” barrier that can significantly slow down the renal clearance of ultrasmall Au NCs with sizes in the sub-nanometer regime (Du et al., 2017).

**FIGURE 5:**
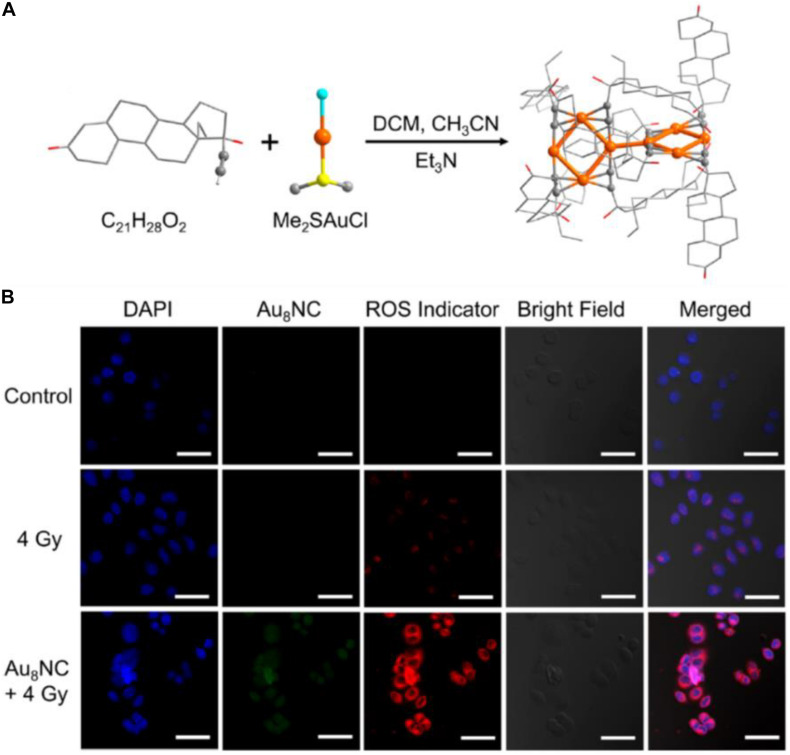
**(A)** Schematic of the synthesis of Au_8_NCs; color codes: orange indicates Au, red indicates O, yellow indicates S, turquoise indicates Cl, and gray indicates C. Hydrogen atoms are omitted. **(B)** Intracellular ROS imaging of EC1 cells at 6 h with different treatments. Reproduced with permission. Copyright 2019, American Chemical Society.

The above cases clearly demonstrate that, compared with larger Au NPs, the ultrasmall Au NCs hold great advantages for kidney filtration and hence can improve renal clearance. Another merit to employ NCs for cancer treatment is that the ligand of the Au NCs can be engineered and the endowed functions of the Au NCs can be correlated with the surface ligand and the metal core. Recently, Jia et al. (2019) reported that an alkynyl-terminated ligand protected Au_8_ nanoclusters (Au_8_NCs) and their radio sensitization for tumor targeting. As presented in Figure 5A, the Au_8_(C_2__1_H_2__7_O_2_)_8_ NCs were synthesized through a one-pot method by reacting C_2__1_H_2__8_O_2_ (levonorgestrel) with Me_2_SAuCl. Au_8_NCs exhibited satisfactory biocompatibility and high luminescent properties with a quantum yield of ∼59%. It is generally believed that the production of ROS upon X-ray irradiation is crucial during radiotherapy, and the ROS burst attacks the covalent bond of DNA, resulting in cell apoptosis and/or cell death. The authors examined the underlying mechanism of Au_8_NCs-mediated radio sensitization by measuring the intracellular ROS levels through confocal imaging. As illustrated in Figure 5B, the average intensity in the experimental group (Au_8_NCs + 4 Gy) was 3.7 times greater than the group without the radiosensitizer, and the control group showed barely red fluorescence. The results solidly verified that the Au_8_NCs were able to catalyze the ROS formation.

**FIGURE 6:**
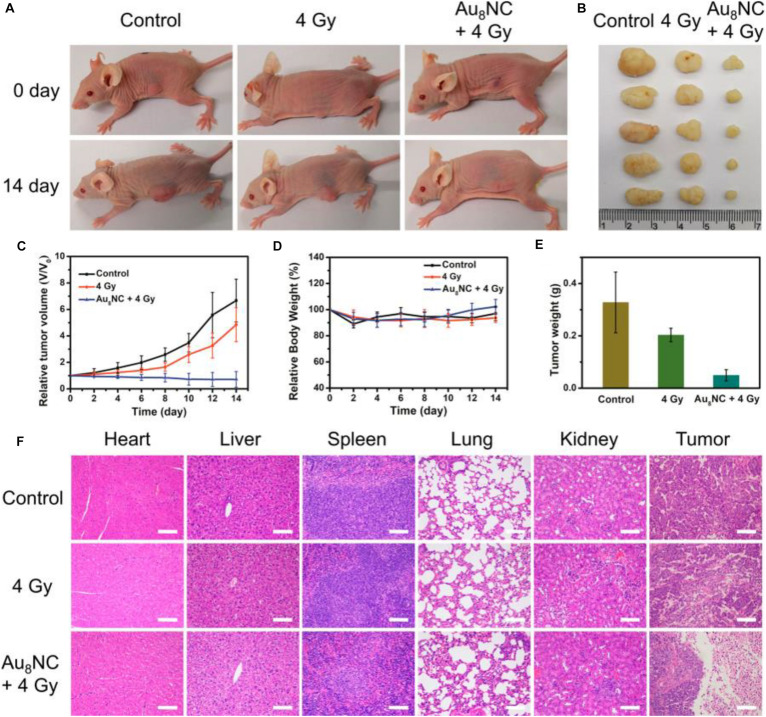
*In vivo* tumorigenicity assay of Au_8_NCs under different conditions. **(A)** Representative images of mice under various conditions at days 0 and 14. **(B)** Images of dissected tumors. **(C)** Relative tumor volume curves of the mice. **(D)** Relative mouse body growth curves. **(E)** Statistical results of the tumor weights. **(F)** H&E histological staining of excised organs and tumor slices. Reproduced with permission. Copyright 2019, American Chemical Society.

The radio sensitization effect of the Au_8_NCs were then assessed *in vivo* by a tumor assay (Jia et al., 2019). The tests with a control group and phosphate-buffered saline (PBS)-treated group were also conducted as comparison. After the cells were injected into the mice and treated with different doses, the tumor size and body weight were monitored every day (Figures 6A–E). The tumor volumes in the control group increased about 5 times, however, the tumor volumes in the Au_8_NCs + 4 Gy group drastically reduced. In addition, the relative body weights of the mice under different conditions remained almost unchanged over 14 days, suggesting low toxicity *in vivo*. Furthermore, hematoxylin and eosin staining of the tumors and organs was conducted to evaluate the treatment. Widespread damage was observed in the tumor tissue from the Au_8_NCs + 4 Gy group compared to the other two groups, and no histopathological abnormalities in the organs were observed (Figure 6F). These findings demonstrated the significantly enhanced tumor-suppressing efficacy of the Au-NCs-based sensitizer. Note that, in the Au_8_NC, Au exhibited (I) valence without the presence of Au(0) species, that is, Au_8_NC is a complex rather than a cluster, so if the size becomes larger, can the Au(0) species in the NCs affect the radio-sensitizing properties? To answer this question, further study might be necessary. Another interesting point for this investigation is that the ligand of levonorgestrel itself is a drug, hence Au_8_NCs worked very well and can be highly stable. Introducing drug-based ligands might represent a future path for the development of atomically precise Au NCs as efficient radiosensitizers.

## Challenges and Outlooks

In summary, we have given a brief review of ultrasmall Au and Ag NCs for biomedical applications, with particular emphasis on bioimaging and biosensing, anti-microbial applications, and tumor targeting and cancer treatment. The unique properties of the Au and Ag NCs, including their ultrasmall size, excellent photoluminescence, post-functionalized surface chemistry, and outstanding bio-compatibility, mean they hold great potential for use in biomedical applications.

Although significant progress has been made, some critical challenges still remain to be resolved in future. These challenges can be listed as follows:

1. The targeting specificity issue. When using Au/Ag NCs for *in vivo* study, especially for therapeutics or diagnostics such as cancer treatment, the specificity must be enhanced. Although the surface capping ligand of NCs can be modified, low-cost and facile methods to tailor the ligands are still quite limited.
2. The comprehensive fundamental study of Au and Ag NCs in a biological environment is lacking. For *in vivo* study, the biological environment is very complex, and it still lacks the fundamental deep understanding on the interactions between Au/Ag NCs and the biological units. For instance, the effects of Au/Ag NCs on cell growth and living microorganisms over prolonged periods of time must be extensively and intensively studied. The systematic study also needs to be carried out to clarify the effects of the functionalities of Au/Ag NCs on their targeting, biodistributions, and toxicity in the complicated biological set-up.
3. The toxicity issue is still a big concern. Even if some previous reports have shown that most ultrasmall Au NCs can be cleared out of the body through the urinary system, the toxicity of the residual Au NCs still can be a problem when they are engineered by therapeutics and diagnostics *in vivo*. Ag NCs would be more complicated, as it may decompose to form Ag(0) substance, which imposed additional challenges for further study. The bio-nanotoxicity investigations are currently flourishing globally, but more attention is needed for biofunctionalized Au and Ag NCs.

As Au and Ag NCs possess different physiochemical properties, when engineering them for biomedical applications, they have some different specific challenges. For instance, Ag NCs have excellent anti-microbial capabilities but their stability is an issue, as Ag NCs can’t be preserved for a prolonged time, such as a few months, without decomposition. However, for Au NCs, they are robust and some of them can be stable for over several months, but their anti-microbial properties are not as impressive as Ag NCs. Therefore, improving the stability of Ag NCs or synthesizing more stable Ag NCs should be a further direction, while enhancing the anti-microbial capabilities of Au NCs is also promising in the regime of using metal NCs for anti-microbial applications.

These shared and specific challenges of Au and Au NCs indicate great opportunities; future research efforts are encouraged to resolve the above-mentioned challenges. Resolving these challenges forms part of the outlook in this rapidly booming field. Besides that, some other research directions also deserve more focus. Specifically, the additional outlooks include:

1. The catalytic properties for Au and Ag NCs can be potentially exerted. It is well known that Au/Ag NCs possess extraordinary catalytic properties in various chemical reactions (Li and Jin, 2013; Tang et al., 2018; Du et al., 2020), but how to engineer their catalytic properties for biomedical processes still needs to be explored.
2. It holds great potential to integrate ultrasmall Au and Ag NCs with other biomolecules/biomaterials to function synergistically. Au and Ag NCs have some unique advantages, but they also possess some drawbacks that might be difficult to overcome. With the development of biological science and modern medicine, precise therapeutic or diagnostic studies are highly demanding. Although some advances have been made to couple Au and Ag NCs with other biomolecules/biomaterials, they is still much left to be developed to realize the “precise medicine.”

Overall, ultrasmall Au/Ag NCs have some attractive features which are not available from the larger NP counterparts. With the rapid developments of medical techniques and nanotechnology, we anticipate more opportunities will be open up for Au/Ag NCs in biomedical applications.

## Author Contributions

JT, HS, and GM did the literature search and wrote the original manuscript. JT, LL, and ZT conceived the idea and provided financial support. LL and ZT polished the final manuscript. All authors contributed to the article and approved the submitted version.

## Conflict of Interest

The authors declare that the research was conducted in the absence of any commercial or financial relationships that could be construed as a potential conflict of interest.
